# Thyroid gland hemorrhage after blunt neck trauma: case report and review of the literature

**DOI:** 10.1186/s12893-017-0322-y

**Published:** 2017-11-28

**Authors:** Johannes Lemke, Markus N. Schreiber, Doris Henne-Bruns, Gregor Cammerer, Andreas Hillenbrand

**Affiliations:** 1 Department Surgery, Division of General and Visceral Surgery, Ulm University Medical Center, Albert-Einstein-Allee 23, 89081 Ulm, Germany; 2 Department of Anesthesiology, Ulm University Medical Center, Albert-Einstein-Allee 23, 89081 Ulm, Germany

**Keywords:** Blunt neck trauma, Thyroid gland hemorrhage, Surgery

## Abstract

**Background:**

Thyroid hemorrhage is considered to be an uncommon complication following blunt trauma to the neck. This condition is potentially life-threatening due to airway compression and may therefore require emergency airway management and surgical intervention in some cases.

**Case presentation:**

We present the case of a 52-year-old woman who experienced a traumatic thyroid gland rupture (right lobe) with subsequent active arterial bleeding from branches of the inferior thyroid artery. On the same day, the patient presented to our emergency department with a painful swelling of the neck with an inspiratory stridor and hoarseness a few hours after a cycling accident. A right hemithyroidectomy was performed. The postoperative course was uneventful. We identified 33 additional cases published in English literature within the last 30 years, reporting blunt trauma to the neck with hemorrhagic complication of the thyroid gland. We provide a systematic review and particularly consider the aspects of endocrine surgery.

**Conclusion:**

The treatment approach for patients with blunt thyroid trauma should be dependent on the extent of the thyroid injury. Patients with tracheal compression, active bleeding and increasing hoarseness/shortness of breath require emergency airway control and often surgical exploration for hemorrhage control followed by resection of the ruptured thyroid. Importantly, in contrast to routine thyroid surgery, no electromyographic endotracheal tube is used during emergency intubation. Exchange of an endotracheal tube should be carefully evaluated due to difficult airway management in this setting. For protection against double-sided recurrent nerve palsy and postoperative hypoparathyroidism, a unilateral approach is preferable whenever possible.

## Background

Thyroid hemorrhage is a rare injury in patients suffering from blunt trauma to the neck. However, it is considered a potentially life-threatening condition due to airway compression. An expanding hematoma of the thyroid compartment may compress the trachea leading to respiratory distress, demanding prompt airway management and/or surgical intervention in some cases. Isolated trauma to the thyroid is very rare and there are only a few reports in the English literature. We present a case of a young woman with a thyroid gland hemorrhage after blunt neck trauma which required emergency airway management and surgical intervention. In addition, we provide a systematic review of published reports of patients suffering from traumatic thyroid gland hemorrhage and particularly consider the aspects of airway management and endocrine surgery. Based on these considerations, we outline therapeutic strategies including surgical and non-surgical approaches to thyroid gland hemorrhage.

## Case presentation

**Fig. 1:**
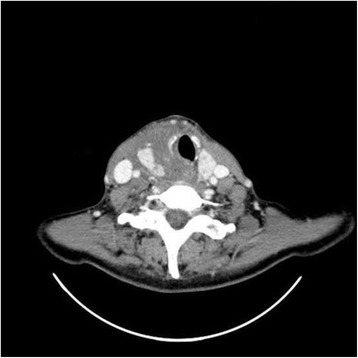
Contrast-enhanced computed tomography revealed a laceration of the right thyroid lobe with diffuse hematoma

A 52-year-old woman experienced a blunt neck trauma due to a bicycle accident. After a car collision car the impact with the windscreen wipers caused the injury. At the time, the patient was suffering from Graves’ disease, positive for anti-thyroid-stimulating hormone receptor antibodies, with a multinodular goiter and was receiving long-term medical therapy with carbimazole 5 mg per day. Two hours after the accident, the patient presented to the emergency department with painful swelling of the neck, inspiratory stridor and increasing hoarseness. The clinical examination revealed a palpable mass on the right anterior part of the neck. Emergency tracheal intubation was performed due to progressive breathlessness and dyspnea. Subsequently, a contrast-enhanced computed tomography revealed a laceration of the right thyroid lobe with surrounding hematoma and tracheal deviation to the contralateral side (Fig. 1). Therefore, the patient underwent emergency neck exploration with a 4-cm Kocher incision followed by a right hemithyroidectomy. Intraoperatively, the dorsal aspect of the right lobe of the thyroid gland was found to be ruptured with active arterial bleeding from branches of the inferior thyroid artery. The recurrent laryngeal nerve was macroscopically identified and preserved. Because of the bleeding into the surrounding tissue, it was particularly difficult to definitely identify the parathyroid glands due to anatomical complexity in this case. Even the location of the upper parathyroid gland, normally identified on the posterior surface of the upper pole of the thyroid gland*,* was not obvious, due to disturbed surrounding tissue caused by the bleeding*.* Postoperatively, the patient was monitored in the intensive care unit for 32 h. The patient was extubated after a further six hours, and the subsequent postoperative course was uneventful. Hoarseness disappeared spontaneously after two days. The histopathological analysis confirmed a multinodular goiter. Laryngoscopy was performed three days postoperatively and did not indicate any pathological alterations. Thyroid hormone levels were in the normal range directly after surgery and three weeks postoperatively. The one-month postoperative follow-up revealed normal thyroid function, consistent with full recovery of the patient.

## Review of the literature

To identify additional cases of thyroid gland hemorrhage after blunt neck trauma a Medline search was performed using the terms “thyroid rupture”, “traumatic thyroid”, “thyroid trauma” and “thyroid hematoma”. Subsequently, non-English publications as well as manuscripts published before 1986 were excluded from further analysis. All remaining publications were screened and manuscripts relevant for the current study were selected for further analysis. We identified 32 additional cases published in English literature within the last 30 years reporting blunt trauma to the neck with hemorrhagic complication of the thyroid gland (Table 1). 19 patients were female (58%). Median age was 44 years (min 8; max 89). Mechanisms of trauma included bicycle, car and motorcycle accidents, falls and sports accidents. In eight patients emergency airway management was required due to respiratory distress and insufficiency. Half of all patients (*n* = 17) had an established diagnosis of pre-existing thyroid disease or this was detected postoperatively. In 16 patients (48%) a surgical approach was required. In one of those patients ligation of an artery was sufficient. However, in most patients a hemithyroidectomy was performed and in a subset of patients a total thyroidectomy was pursued (4 out of 16; 25%).

**Table 1:** Thirty four reported cases in English literature within the last 30 years on blunt trauma to neck leading to hemorrhage of the thyroid gland

Author	Gen.	Age	Trauma	Symptoms	AM^a^	Path^b^	Surg^c^
Ahrens, 2012 [8]	F	75	Bicycle	Swelling, Dyspnea	Yes	No	Yes [HT]
Arana-Garza, 2015 [9]	F	19	Sports	Neck Pain, Dysphagia	No	No	Yes [HT]
Behrends, 1987 [10]	M	30	Horse	Swelling	No	Yes	No
Blaivas, 1998 [4]	M	34	Car Accident	Swelling, Dysphagia, Dyspnea	No	Yes	Yes [TT]
Chartier, 2010 [5]	F	75	Fall	Swelling, Dysphagia, Dyspnea	No	Yes	Yes [HT]
Donatini, 2011 [11]	F	53	Fall	Dyspnea, Hoarseness	Yes	yes	Yes [TT]
Hagiwara, 2007 [12]	M	20	Motorcycle Accident	n/a	Yes	No	No
Hamid, 2004 [13]	M	13	Motorcycle Accident	Hoarseness	No	No	No
Hara, 2016 [14]	F	71	Fall	Neck Pain	No	No	Yes [HT]
Heizmann, 2006 [3]	F	47	Car Accident	Hoarseness, Swelling	No	No	Yes [HT]
Hishoren, 2004 [15]	F	30	Car Accident	Neck Pain, Dysphagia	No	Yes	No
Hsieh, 2000 [16]	F	25	Car Accident	Dyspnea, Swelling	Yes	No	No
Lemke, 2017	F	52	Bicycle	Dyspnea, Swelling	Yes	Yes	Yes [HT]
Lodder, 2001 [17]	F	34	Sports	n/a	No	Yes	No
Oertli, 1994 [18]	F	82	Fall	Swelling, Respiratory Distress		Yes	Yes [TT]
Oka, 2007 [19]	F	65	Fall	Dyspnea	Yes	Yes	Yes [HT]
Park, 2006 [1]	F	26	Fall	Neck Pain	No	No	No
Pérez Fontán, 2001 [20]	F	44	Assault	Neck Pain, Hematoma	No	n/a	No
Pop, 2005 [21]	F	76	Car Accident	Dyspnea	Yes	Yes	Yes [HT]
Schipper, 2008 [22]	F	60	Fall	Swelling, Dyspnea	No	Yes	Yes [HT]
Rivera-Serrano, 2012 [23]	M	46	Sports	Neck Pain, Swelling, Dysphagia, Dysphony	No	Yes	No
Saylam, 2009 [24]	F	69	Fall	Hoarseness	No	No	No
Shin, 2015 [25]	M	24	n/a	n/a	n/a	No	Yes [HT]
Shin, 2015 [25]	M	8	n/a	n/a	n/a	Yes	No
Sow, 2013 [26]	M	41	Motorcycle Accident	Pain, Dyspnea, Swelling, Hoarseness	No	No	Yes [HT]
Stunell, 2006 [27]	M	35	Bicycle	Swelling	No	No	No
Tam, 2016 [28]	M	51	Hit by Waterjet	Swelling, Dysphagia	No	Yes	No
Tsukahara, 2016 [29]	F	89	Traffic Accident	Respiratory Distress, Severe Dyspnea	n/a	Yes	Yes [Lig]
Ved, 2016 [30]	M	42	Assault	Respiratory Distress, Swelling	Yes	Yes	Yes [TT]
Vora, 2001 [31]	M	21	Car Accident	Swelling	No	Yes	No
Watson, 1999 [32]	M	23	Assault	Swelling, Dysphagia	No	n/a	No
Weeks, 2005 [2]	F	50	Car Accident	Neck Pain, Hoarseness Dysphagia, Swelling	No	No	No
Zawawi, 2013 [33]	M	13	Sports	Neck Pain	No	No	No

*HT* hemithyroidectomy, *TT* total thyroidectomy, *Lig.* arterial ligation

^a^AM = airway management was required

^b^Path = pre-existing pathological alteration of the thyroid gland was known or detected during surgery

^c^Surg = Surgery was necessary due to thyroid hemorrhage (Yes vs. No) and extent of surgery is shown in square brackets

We found no correlation between the type of trauma, age of patients, onset of symptoms and the extent of surgery. Nearly half or authors report a delayed onset of symptoms, even later than 24 h after initial trauma. Postoperative complications are rarely reported and were mostly transitional hoarseness, while many authors do not report on the performance or results of postoperative laryngoscopy.

## Discussion and conclusions

A number of case reports and case series address thyroid gland rupture and hemorrhage following blunt neck trauma. Many of the reported cases have involved rupture of a pre-existing goitrous thyroid gland [1]. Painful pre/paratracheal swelling, airway obstruction and dyspnea due to a large hemorrhage are the most common symptoms after thyroid gland rupture. Thyroid rupture is treated either surgically or conservatively. About half published cases of traumatic thyroid hemorrhage were surgically treated. Surgical intervention specifically is determined based on evacuation of the rapidly extending hematoma. Especially in severe respiratory distress, airway control alone seems not to be sufficient and emergency operative surgery should be favored. Other management strategies include overnight observation or conservative treatment with close airway monitoring [2]. Importantly, many authors report a delayed onset of symptoms, even later than 24 h after initial trauma [3–5]. Therefore, despite rarity of the condition and the highly individual course of disease, duration of monitoring should last longer than 24 h. When a surgical approach is pursed, simple ligature of the affected blood vessel appears to be unfeasible in many cases due destroyed thyroid tissue. Therefore, most reports describe a (hemi)thyroidectomy when surgery was performed. We decided to immediately perform surgery because the patient presented with inspiratory stridor and increasing hoarseness followed by progressive breathlessness and dyspnea. These severe symptoms are potentially life threatening and are typically also observed as a sign of postoperative hemorrhage after routine thyroid surgery. Hematomas in the cervical compartment can be lethal if they result in airway compression [6]. In this respect it is interesting to note that the trachea is more stable to frontal pressure. In contrast, lateral compression quickly leads to tracheal compression. Moreover, parathyroid glands of the affected side are at risk since perithyroid hematoma could hinder their identification. In rare cases, a thyroid gland injury due to blunt neck trauma is complicated by thyrotoxicosis in patients with pre-existing hyperthyroidism [6, 7]. Therefore, thyroid hormones should be measured immediately and regularly after blunt neck trauma.

Today, elective thyroid surgery is commonly carried out using an electromyographic endotracheal tube to monitor the recurrent laryngeal nerves’ functions. In the case of emergency airway management, an electromyographic tube is usually not available. Consequently, in the cases where surgery is required after emergency intubation, nerve monitoring is not feasible. Therefore, in an emergency setting, a total hemithyroidektomy should be avoided unless absolutely necessary to prevent bilateral paralysis of the recurrent nerve. Moreover, we suggest that tube exchange to an electromyographic tube should not be routine procedure. Tube replacement could be hampered by an increased thyroid hematoma. In the absence of an endotracheal tube, electromyography using a needle electrode could be an alternative if a needle electrode is on hand and handling is familiar.

**Fig. 2:**
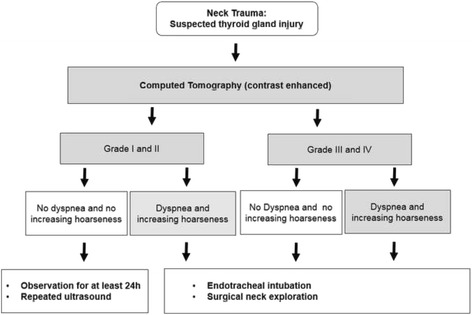
Algorithm for management of thyroid gland injury (based on the algorithm proposed by Heizmann et al.). Classification of Blunt Thyroid Injuries Grade Description of the Lesion by Heizmann: I Small parenchymal lacerations, bleeding into nodules, subcapsular hematoma. II Rupture of the thyroid gland with / without parathyroid hematoma. III Rupture of the thyroid gland with significant neck hematoma including tracheal compression. IV Rupture of the thyroid gland and neck hematoma with associated lacerations to the larynx skeleton and/or to carotid and jugular vessels

Heizmann et al. proposed a classification of blunt thyroid injuries and a treatment algorithm [5]. A small parenchymal laceration is staged as grade I injury, a rupture with a parathyroid hematoma or a neck hematoma as grade II and III, respectively. An additional laceration of surrounding tissue corresponds to grade IV injury. The treatment algorithm suggests a contrast-enhanced computed tomography for the diagnosis. It also recommends that grade I and II injuries are managed with overnight hospital observation. In contrast, emergency tracheal intubation with operative neck exploration is suggested for grade III and IV injuries. The algorithm is based purely on radiological findings. Symptoms such as dyspnea and/or increasing hoarseness are not considered in this protocol. However, dyspnea commonly indicates tracheal compression due to an expanding hematoma and requires emergency airway management. Moreover, hoarseness is a sign of pressure-mediated injury of the recurrent laryngeal nerve which may cause irreversible nerve damage unless there is immediate surgical intervention. Therefore, we suggest that criteria for decision-making in patients presenting with thyroid injuries need to be expanded according to symptoms, most importantly dyspnea and increasing hoarseness. We therefore propose a novel algorithm based on the one suggested by Heizmann et al. including these criteria (Fig. 2). In summary, the initial treatment approach for patients should be dependent on the extent of the thyroid injury determined by CT scan and clinical symptoms. Parenchymal hematoma without significant neck hematoma should be observed in intensive care for at least 24 h due to imminent risk of airway compression. Patients with significant neck hematoma including tracheal compression, active bleeding and aggravated shortness of breath and/or hoarseness require emergency airway control and immediate surgical exploration for hemorrhage control followed by partial resection of the ruptured thyroid. Exchange of an endotracheal tube should be carefully evaluated due to difficult airway management in this setting. For protection against double-sided recurrent nerve palsy and postoperative hypoparathyroidism, a unilateral approach is preferable whenever possible.
